# Determinants of Health Care-Seeking Delay among Tuberculosis Patients in Rural Area of Central China

**DOI:** 10.3390/ijerph15091998

**Published:** 2018-09-13

**Authors:** Yeqing Tong, Xuhua Guan, Shuangyi Hou, Li Cai, Yadong Huang, Lei Wang, Faxian Zhan, Yuqin Shi, Jiafa Liu

**Affiliations:** 1 Institute of Infectious Diseases, Hubei Center for Disease Control and Prevention, Wuhan 430079, China; yxx@hust.edu.cn (Y.T.); g_xuhua@21cn.com (X.G.); h_shuangyi@aliyun.com (S.H.); huangyadong12@163.com (Y.H.); w_lei@aliyun.com (L.W.); zhfxian@outlook.com (F.Z.); 2 School of Public Health, Huazhong University of Science and Technology, Wuhan 430071, China; 3 Wuhan Center for Disease Control and Prevention, Wuhan 430015, China; whnip@whcdc.org; 4 School of Health Sciences, Wuhan University, Wuhan 430071, China; 5 School of Public Health, Wuhan University of Science and Technology, Wuhan 430081, China

**Keywords:** tuberculosis, health care-seeking delay, risk factors

## Abstract

*Background* The prevalence of tuberculosis (TB) in low and middle-income countries is a significant public health and social concern. TB is a common infectious disease caused by the *Mycobacterium tuberculosis* infection, which has a widespread infection rate. Health care-seeking delay maybe one of the most important neglected risk factors for the spread of TB. *Objectives* The aim of this study was to understand the situation of health care-seeking delay among rural tuberculosis patients in Hubei Province, and explore its risk factors. *Methods* A total of 1408 rural tuberculosis patients were surveyed using a standard structured questionnaire in three cities of Hubei Province during the past two years. *Results* For the 1408cases of pulmonary tuberculosis, 39.70% of them were health care-seeking delayed. Logistic regressions indicate that the Han nationality, farming careers, the over 45 min walk to the township’s hospital, and awareness of the national TB free treatment policy, were significantly associated with higher odds of a delay in care seeking. *Conclusions* The prevalence of health care-seeking delay among tuberculosis patients was high in rural areas. It is essential to take comprehensive targeted interventions to reduce care-seeking delay.

## 1. Introduction

Tuberculosis (TB), a respiratory infectious disease which has existed for millennia, remains a serious global health and social problem. China ranks as the third highest among the 22 highest TB burden countries in the world. China records about 1,000,000 TB cases every year [1], with an estimated prevalence of 459/100,000 [2] for all forms of TB in total. The majority of cases are in rural areas. Timely diagnosis and treatment of TB are beneficial for TB control and prevention [3,4]. The main target of TB control and prevention is to detect and treat new incident cases as early as possible. However, the target is hard to meet because patients usually go to the health facility late and health care staff may fail to diagnose TB in populations with early classic symptoms [4]. The delays in health care seeking for TB related symptoms are neglected due to both patient and provider factors. A cross-sectional study performed in three provinces in China indicated that average monthly working time and hemoptysis or bloody sputum were significantly associated with delayed initial health care seeking [5]. A recent systematic review which included 10 studies revealed that some socio-demographic factors (including age, gender, and education level) and accessibility of medical institutions were linked with a delay in health care-seeking [6]. In addition, studies at Zambia and Ethiopia found that HIV infections status and getting treatment from holy water or traditional healers were related to delayed health care-seeking [7,8]. The TB patients’ health care-seeking behavior has been proved to be affected by the economic factors in various countries [9,10,11,12,13,14]. Delays of health care provider side in diagnosis and treatment are also recorded in African Asian countries [7,15].

The phenomenon of delay in diagnosis and treatment, which are the important factors for the treatment failure of TB patients, is particularly pronounced in rural areas of China [2,4]. However, the neglected delay risk factors in rural areas have not been well studied in China. The purpose of this study was to investigate the health care-seeking behavior of TB patients, and explores the degree of treatment delay and its influencing factors in rural areas of China, thus to provide a scientific basis for China and other developing countries to reduce health care-seeking delay, increasing detection rate, and further optimize the TB prevention and control system.

## 2. Material and Methods

### 2.1. Study Location

Hubei Province, with a population of nearly 60 million, is located in Central China. It is a very representative of economy, politics, culture, and geography in China. According to the economic status and geography of various cities in Hubei Province, the three representative areas of Xiangyang City, Huanggang City, and Enshi City were selected as the research sites, and then one hospital was randomly selected from each of the three cities. The participants of this study met the following three criteria: (1) TB patients who lived in rural areas and were registered in the local professional institutions for TB control in the past two years; (2) TB patients who had no psychiatric illnesses; and (3) TB patients who were willing to participate in the study. Finally, 1408 questionnaires were included in the statistical analysis.

### 2.2. Data Collection

Data were collected by trained doctors of TB control institutions using a standard structured questionnaire which was developed based on a review of the published literature [16,17,18,19]. Risk factors associated with health care-seeking delay were collected through interviews. Before the start of the formal study, a pilot study which included 19 male TB patients and 16 female TB patients was conducted to ensure that the questions were clear and understandable to all participants. The questionnaire contained information on the socio-demographic characteristics of TB patients, walk time to the nearest town hospital, previous TB treatment, and awareness of the relevant national policy.

### 2.3. Judgment Criteria of Health Care-Seeking Delay

According to China’s Ministry of Health TB prevention and control work manual, if the interval from the emergence of the cough, expectoration, and other symptoms of TB to the first doctor visit is more than 2 weeks, the case will be defined as a health care-seeking delay in the research [20,21,22,23,24,25].

### 2.4. Statistical Analysis

The data were double inputted by Epidata 3.0 (EpiData Association, Odense, Denmark) to accurately set up a database, and then is studied by descriptive analysis and logistic regression analysis with SAS9.0 (SAS Institute Inc., Cary, NC, USA). Before statistical analysis, Markovchain Monte Carlo (MCMC, Tree age Inc., Williamstown, MA, USA) was used to conduct multiple imputations of missing data.

### 2.5. Ethics Statement

Our study was approved by the Institutional Review Board of Hubei Center for Disease Controls and Prevention (2017ADC061), and signed informed consents were obtained from all participants or from patients’ representatives if direct consent could not be obtained. The experiment methods were carried out in accordance with the approved guidelines and regulations, and all experimental protocols were approved by the Institutional Review Board of the Hubei Center for Disease Controls and Prevention.

## 3. Results

### 3.1. Descriptive Analysis of Basic Situation of TB Patients

The study investigated 1408 TB patients, with an average age of 47.59 (SD = 16.90) years old. The majority of the TB patients are male, accounting for 67.54%. Their average monthly income is 1970.08 yuan, of which a monthly income of less than 800 yuan accounts for 19.74%. In addition, the average time required for the patients to reach the nearest township hospital was31.38 (SD = 27.91) min. Among the 1408 TB patients, 559 cases were delayed in health care seeking, accounting for 39.70%. The specific data are illustrated in Table 1.

### 3.2. Analysis of Factors Affecting the Health Care-Seeking Delay of TB Patients

Univariate logistic regression analysis revealed that there were obvious correlations between the health care-seeking delay and the work status, average monthly income, distance to the nearest town hospital, and awareness of the relevant national policy, while other factors have no significant correlation with the health care-seeking delay. Multivariate logistic regression analysis further showed a significant increase in the risk of delayed health care-seeking for patients with Han nationality, farming careers, a distance of over 45 min to the nearest town hospital, or not knowing the relevant national policy, by controlling other factors, while other factors had no significant correlation with health care-seeking delay. The specific data is illustrated in Table 2.

## 4. Discussion

There are many studies on the health care-seeking delay for TB patients in the Asian, African, and European countries, and the results differ widely in different regions, because of different research objects and methods. Studies have shown that the delay rate ranges from 32.6% to 67.87% [26,27,28,29,30]. Our current study found that the delay rate in the rural areas Central China was 38.80%, less than some areas, but generally still in a high level. Thus, it is necessary to take efficient actions to encourage TB patients to seek medical help promptly.

Studies have found that the social demographical factors were the main factors influencing the treatment delay of TB patients [31,32,33,34,35]. Univariate logistic regression analysis showed that work status, income, walk time to the nearest town hospital, and awareness of the relevant national policy were associated with a delay in health care seeking. When adjusting for other factors, ethnicity, occupation, walk time to the nearest town hospital, and having knowledge of the national TB free treatment policy were approved to be risk factors. In our study, we first found that the risk of health care-seeking delay in Han patients and farming patients was extremely high. The non-farming population refers to workers, employees of enterprises, self-employed businessmen, and so on. Compared with this group, farming TB patients were less likely to acquire knowledge and information about TB prevention due to the limitations of their living and working conditions. Moreover, their medical behavior was largely influenced by family economy factors, which might be related to the increased risk of health care-seeking delay. An acceptable theory to explain the increased risk of health care-seeking delay in Han patients, however, is still not agreed upon and needs further study. In addition, the results confirmed the key role of health care accessibility in TB control. Patients who went to medical institutions more than 45 min away were more prone to experience delay in health care-seeking, which suggested that the government should pay more attention to remote rural areas and appropriately increase the allocation of medical resources in rural areas. Moreover, patients who did not know the national TB free treatment policy were proven to be associated with health care-seeking delay. To reduce health care-seeking delay and strengthen TB control, the government should strengthen policy publicity and expand the scope of the free treatment policy. The strengths of our current study are a relatively large sample size in the Hubei province of China and that TB patients were diagnosed by the medical staff of local professional institutions. Sputum smear microscopy was performed first, and if the sputum smear was negative or TB was clinically suspected, a chest X-ray test was carried out. Rigorous methods were applied to ensure that all the examination procedures complied with clinical guidelines. Our study also has some limitations. First and foremost, it is a retrospective study and a recall bias could not be completely excluded for the group of patients with TB. Secondly, the results could be influenced by some confounding factors, such as family support, social stigma to TB patients, and other unexpected factors. Thirdly, the participants included in this study were TB patients who had been registered in the local professional institutions. Patients without registration might have longer delays in health care seeking. Therefore, selection bias might exist in our study. Finally, it is possible that our findings might be applied only to the Chinese rural population [35].

## 5. Conclusions

Taken together, we found that the Han nationality, average monthly income, and the time needed to reach the nearest town hospital have significant effects on health care-seeking delay and proved that the accessibility of health services may affect health care-seeking behavior [36,37,38]. However, we did not find a strong link between sex, age, and literacy, and treatment delay, somewhat at variance with other studies [39,40,41,42,43,44]. It is worth noting that we should strengthen TB control in remote areas, particularly the areas with unsound medical systems. Moreover, 38.52% of the TB patients do not know the national policies, suggesting the necessity of strengthening the promotion of TB prevention policies and knowledge. Therefore, it is imperative for us to take drastic measures to improve the health care-seeking delay situation in Chinese rural areas from our discovered determinants in the study, especially those easily neglected determinants. In general, these findings provided the first evidence for China and other high-burden countries with TB to formulate effective TB control policies.

## Figures and Tables

**Table 1:** The descriptive analysis of the influencing factors of health care-seeking delay for tuberculosis (TB) patients.

Variables	Total (*n* = 1408)		Non-Delayed (*n* = 849)		Delayed (*n* = 559)		*p*-Value
	*n*	%	*n*	%	*n*	%	
Age (years old)							
<30	300	21.31	192	22.61	108	19.32	0.3983
30~45	247	17.54	150	17.67	97	17.35	
45~60	472	33.52	283	33.33	189	33.81	
>60	389	27.63	224	26.38	165	29.52	
Gender							
Male	951	67.54	571	67.26	380	67.98	0.7768
Female	457	32.46	278	32.74	179	32.02	
Ethnicity							
Han nationality	1016	72.16	603	71.02	413	73.88	0.2419
National minority	392	27.84	246	28.98	146	26.12	
Literacy							
Primary school or below	596	42.33	339	39.93	257	45.97	0.1505
Junior high school	570	40.48	358	42.17	212	37.92	
High school/Technical secondary school	206	14.63	128	15.08	78	13.95	
College or above	36	2.56	24	2.83	12	2.15	
Marital status							
Unwed	241	17.12	156	18.37	85	15.21	0.2474
Married	1104	78.41	658	77.50	446	79.79	
Divorced/widowed	63	4.47	35	4.12	28	5.01	
Occupation							
Farming	1058	75.14	606	71.38	452	80.86	<0.0001
Non-farming	350	24.86	243	28.62	107	19.14	
Medical insurance							
No	74	5.26	40	4.71	34	6.08	0.2594
Yes	1334	94.74	809	95.29	525	93.92	
Average monthly income (yuan)							
<800	278	19.74	144	16.96	134	23.97	0.0011
800~1500	302	21.45	187	22.03	115	20.57	
1500~2500	415	29.47	243	28.62	172	30.77	
>2500	413	29.33	275	32.39	138	24.69	
Treatment							
Initial treatment	1230	87.36	751	88.46	479	85.69	0.1262
Re-treatment	178	12.64	98	11.54	80	14.31	
Walk time to the nearest town hospital (minutes)							
<15	284	20.17	188	22.14	96	17.17	<0.0001
15~30	408	28.98	269	31.68	139	24.87	
30~45	433	30.75	257	30.27	176	31.48	
≥45	283	20.10	135	15.90	148	26.48	
Awareness ofthe relevant national policy							
Yes	914	64.91	574	67.61	340	60.82	0.009
No	494	35.09	275	32.39	219	39.18	

Note: Percentages may not add up to exactly 100%, owing to the rounding off.

**Table 2:** Logistic regression analysis of the influencing factors of health care-seeking delay of TB patients.

Variables	Univariate Logistic Regression Analysis		Multivariate Logistic Regression Analysis	
	OR	95%CI	OR	95%CI
Age (years old) (ref: <30)				
30~45	1.15	0.81–1.63	0.98	0.64–1.51
45~60	1.19	0.88–1.60	0.92	0.61–1.39
>60	1.31	0.96–1.79	0.95	0.61–1.49
Gender (ref: Male)				
Female	0.97	0.77–1.22	0.94	0.74–1.20
Ethnicity (ref: Han nationality)				
National minority	0.87	0.68–1.10	0.68	0.53–0.89 *
Literacy (ref: College or above)				
Primary school or below	1.52	0.74–3.09	0.89	0.40–2.02
Junior high school	1.18	0.58–2.42	0.84	0.39–1.84
High school/Technical secondary school	1.22	0.58–2.58	1.02	0.47–2.23
Marital status (ref: Married)				
Unwed	0.80	0.60–1.08	0.94	0.62–1.42
Divorced/widowed	1.18	0.71–1.97	1.00	0.59–1.72
Occupation (ref: Non-farming)				
Farming	1.69	1.31–2.19 *	1.70	1.23–2.35 *
Medical insurance (ref: Yes)				
No	1.31	0.82–2.10	1.21	0.74–1.98
Average monthly income (yuan) (ref: >2500)				
<800	1.85	1.36–2.53 *	1.36	0.97–1.91
800~1500	1.23	0.90–1.67	0.99	0.71–1.39
1500~2500	1.41	1.06–1.87 *	1.24	0.92–1.66
Treatment(ref: Re-treatment)				
Initial treatment	0.78	0.57–1.07	0.83	0.60–1.15
Walk time to the nearest town hospital (minutes) (ref: <15)				
15~30	1.01	0.74–1.39	1.05	0.76–1.46
30~45	1.34	0.98–1.83	1.30	0.94–1.79
≥45	2.15	1.53–3.01 *	2.16	1.50–3.11 *
Aware of the relevant national policy (ref: Yes)				
No	1.34	1.08–1.68 *	1.28	1.01–1.62 *

* Significantly associated when *p* < 0.05.
